# Synthetic modified *Fezf2* mRNA (modRNA) with concurrent small molecule SIRT1 inhibition enhances refinement of cortical subcerebral/corticospinal neuron identity from mouse embryonic stem cells

**DOI:** 10.1371/journal.pone.0254113

**Published:** 2021-09-02

**Authors:** Cameron Sadegh, Wataru Ebina, Anthony C. Arvanites, Lance S. Davidow, Lee L. Rubin, Jeffrey D. Macklis

**Affiliations:** 1 Department of Stem Cell and Regenerative Biology, Center for Brain Science, and Harvard Stem Cell Institute, Harvard University, Cambridge, Massachusetts, United States of America; 2 Department of Neurosurgery, Massachusetts General Hospital and Harvard Medical School, Boston, Massachusetts, United States of America; 3 Department of Stem Cell and Regenerative Biology, Harvard University, Cambridge, Massachusetts, United States of America; 4 Division of Hematology/Oncology, Program in Cellular and Molecular Medicine, Boston Children’s Hospital, Boston, Massachusetts, United States of America; 5 Department of Stem Cell and Regenerative Biology, and Harvard Stem Cell Institute, Harvard University, Cambridge, Massachusetts, United States of America; Osaka University: Osaka Daigaku, JAPAN

## Abstract

During late embryonic development of the cerebral cortex, the major class of cortical output neurons termed subcerebral projection neurons (SCPN; including the predominant population of corticospinal neurons, CSN) and the class of interhemispheric callosal projection neurons (CPN) initially express overlapping molecular controls that later undergo subtype-specific refinements. Such molecular refinements are largely absent in heterogeneous, maturation-stalled, neocortical-like neurons (termed “cortical” here) spontaneously generated by established embryonic stem cell (ES) and induced pluripotent stem cell (iPSC) differentiation. Building on recently identified central molecular controls over SCPN development, we used a combination of synthetic modified mRNA (modRNA) for *Fezf2*, the central transcription factor controlling SCPN specification, and small molecule screening to investigate whether distinct chromatin modifiers might complement *Fezf2* functions to promote SCPN-specific differentiation by mouse ES (mES)-derived cortical-like neurons. We find that the inhibition of a specific histone deacetylase, Sirtuin 1 (SIRT1), enhances refinement of SCPN subtype molecular identity by both mES-derived cortical-like neurons and primary dissociated E12.5 mouse cortical neurons. *In vivo*, we identify that SIRT1 is specifically expressed by CPN, but not SCPN, during late embryonic and postnatal differentiation. Together, these data indicate that SIRT1 has neuronal subtype-specific expression in the mouse cortex *in vivo*, and that its inhibition enhances subtype-specific differentiation of highly clinically relevant SCPN / CSN cortical neurons *in vitro*.

## Introduction

Subcerebral projection neurons (SCPN) are the broad population of cerebral cortex (cortical) neurons that connect and provide high-level descending control via axonal projections from the neocortex (termed “cortical” here) to distal targets in the brainstem (midbrain, hindbrain) and spinal cord [1–4]. The large subtype of SCPN providing descending motor control to the spinal cord (direct or via sensory feedback) are termed corticospinal neurons (CSN), a term often considered to also include cortical neurons projecting to brainstem targets. SCPN are the brain neurons that degenerate in ALS and related motor neuron diseases, and whose injury (in particular, to CSN) is responsible for the loss of voluntary motor function in spinal cord injury. Early and defining molecular features of SCPN include high-level expression of FEZF2 and CTIP2, required for the specification and control of SCPN molecular, cellular, and anatomical identity [3].

Midway through corticogenesis, post-mitotic SCPN identity is initially masked by transient co-expression of regulators of interhemispheric callosal projection neuron (CPN) development, including SATB2 [2,5–9]. At later stages of maturation, SCPN discontinue expression of SATB2, and further resolve into diverse subpopulations of FEZF2- and CTIP2-expressing projection neurons with cortical area- and target-specific molecular identities, properties, and functional circuit connectivity [10–12]. Multiple epigenetic factors support the post-mitotic identity refinement of contrasting cortical neuron subtypes, such as SCPN and CPN, and enable their maturation [13–18]. Together, these reports suggest that chromatin remodeling might contribute to the identify refinement of ES cell-derived cortical neuron subtypes.

ES/iPSC-based models of cortical differentiation are emerging as useful tools to investigate roles of chromatin modifications in cortical development [19,20]. While protocols for directing cortical differentiation from ES/iPSC cells in systems ranging from monolayer cultures to organoids have succeeded in replicating some of the molecular characteristics of cortical development [21–26], the mature refinement of cortical subtypes is incomplete with these protocols; immature neurons become “stalled” at an mid-embryonic developmental stage [8]. These data suggest that ES-derived cortical cells are unlikely to have a sufficiently permissive molecular context for the precise refinement of SCPN identity. Supporting this concept, inappropriately timed *Fezf2* mis-expression by ES cells does not drive SCPN differentiation [27,28].

We hypothesized that alteration of the chromatin landscape within incompletely specified mouse embryonic stem cell (mES)-derived cortical progenitors might promote a permissive molecular context for *Fezf2*-directed SCPN subtype refinement. To identify candidate chromatin remodeling enzymes, we conducted a high-content screen of mES-derived cortical cells using a library of small molecules that modulate known epigenetic enzymes. Mature SCPN refinement was assessed by measuring changes in the ratio of positive (CTIP2) and negative (SATB2, CTIP1) markers of SCPN differentiation. This strategy emphasizes the utility of multiple exclusionary markers to delineate SCPN-specific differentiation among mES-derived cortical progenitors, in contrast to the approach of evaluating for multiple positive markers that are often expressed in immature SCPN-like neurons.

From this screen, we identify a histone deacetylase, Sirtuin1 (SIRT1), as an effective repressor of *Fezf2*-mediated SCPN molecular refinement. Small molecule inhibitors of SIRT1 (*e*.*g*., EX-527, CHIC-35) enhance *Fezf2*-induced molecular maturation of SCPN by maintaining CTIP2 expression and reducing SATB2 and CTIP1 expression by both mES-derived cortical-like neurons and primary dissociated mouse cortical neurons *in vitro*. We also identify differential refinement of SIRT1 expression in late embryonic cortical neuron subtypes during normal development: elevated SIRT1 expression by CPN, and diminished expression by SCPN. Together, these data identify chromatin remodeling as an important mechanism of cortical subtype refinement both *in vivo* and for *in vitro* directed differentiation, and identify a route to enhanced subtype-specific differentiation of developmentally and clinically important cortical neurons from pluripotent stem cells.

## Results

### ModRNA provided dose- and time-dependent protein expression in mES-derived cells

Synthetic modified mRNA (modRNA) enables precision over gene dosage and timing by multiple cell types [29]. Because modRNA does not integrate into the genome, and has a limited duration of expression (~2 days), modRNA enables transient gene expression without manipulating the genomes of ES-derived cells. ModRNA transfection was evaluated in feeder-free E14Tg2a mES cells undergoing an established monolayer protocol of differentiation that generates heterogeneous, maturation-limited, neocortical-like neurons [8,22,30].

**Fig 1:**
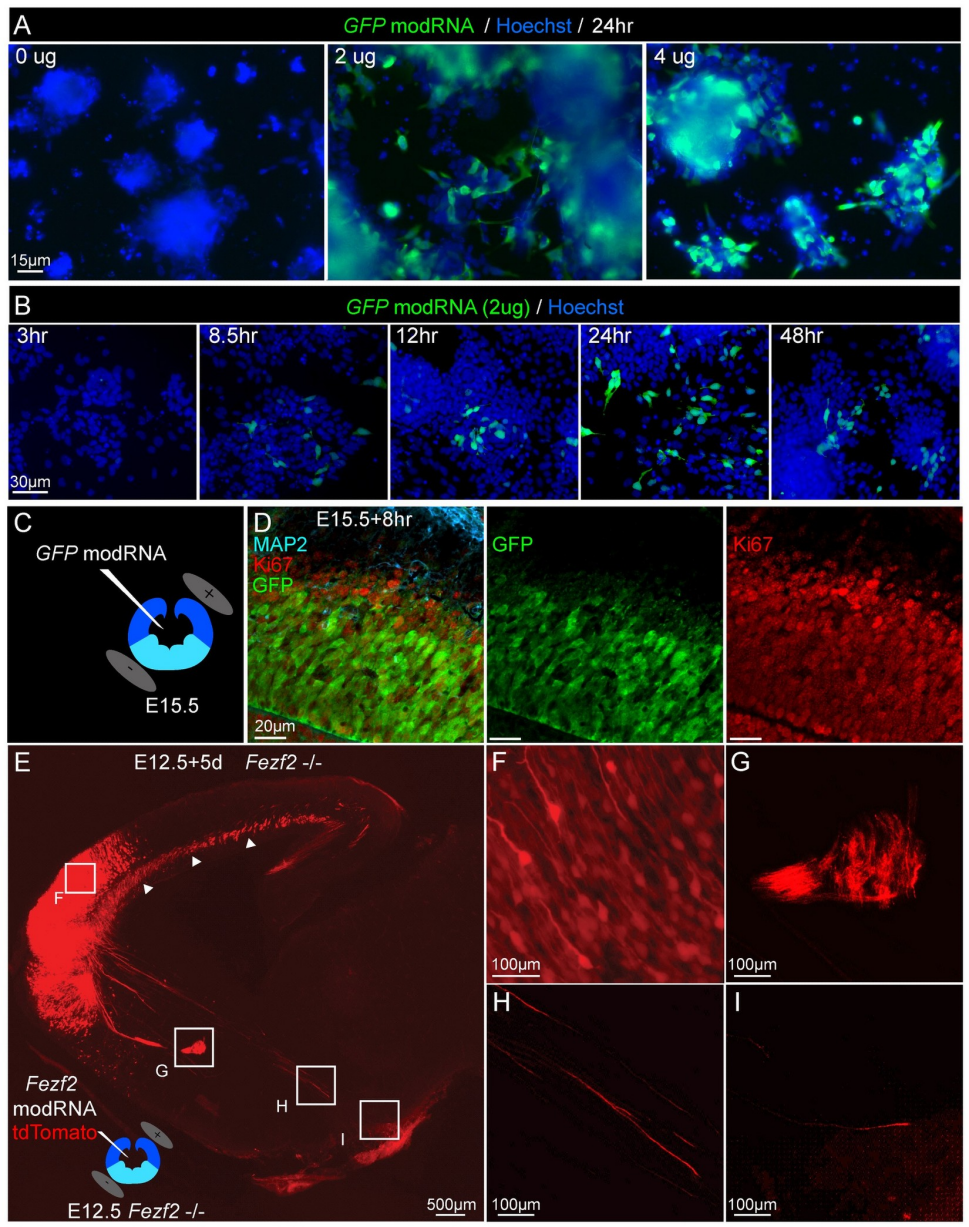
ModRNA enabled temporally controlled, dose-dependent protein expression in cultured mES-derived populations, and *in vivo*. (A) *GFP* modRNA expression was dose-dependent in mES-derived pallial-like progenitors at 14 days *in vitro* (DIV). Native GFP expression was detected in 25–50% of cells after 24hrs; the intensity of expression increased over the range of 0, 2, 4 microgram modRNA transfection. (B) *GFP* modRNA expression was time-dependent over 48hrs. (C) *GFP* modRNA was injected into the lateral ventricles (coronal cross-section of mouse embryo forebrain is shown) and directionally electroporated into the dorsal forebrain / pallium (positive paddle above dark blue colored tissue). (D) Following *in utero* electroporation of *GFP* modRNA (0.8 microgram / microliter), GFP protein (green) is expressed after eight hours, and is restricted to Ki67-expressing progenitors (red) of the pallium, not in more superficially located post-mitotic neurons, which express MAP2 (blue). (E) Sagittal section of an E17.5 *Fezf2*-null mouse following E12.5 *in utero* electroporation of *Fezf2* modRNA (0.8 microgram / microliter) and *tdTomato* plasmid (1 microgram / microliter) showed targeted tdTomato fluorescence appropriately restricted to the rostral pallium. (F) Higher magnification image showing tdTomato expression in the cortical plate. (G) tdTomato+ axons projected across the cerebral commissures, including anterior commissure (depicted) and corpus callosum in (arrowheads in E). (H) Several tdTomato+ axons projected caudal to the thalamus, indicating partial genetic rescue of E12.5 SCPN, which are not present in *Fezf2*-null mice and can only be generated by similar *in utero* rescue experiments [32]. (I) A small subset of these tdTomato+ axons reached the cerebral peduncles (N = 3), confirming partial rescue of SCPN axonal projections.

In agreement with the prior literature, we found a dose-dependent intensity of GFP expression after transfection of mES-derived cells with *GFP* modRNA. At the peak of pallial-like differentiation at day 14, modRNA-induced GFP expression peaked between 12–24 hours, with a sharp reduction of expression by 48 hrs (Fig 1A and 1B). We also found that modRNA transfection is not biased to a specific neural population; modRNA broadly transfected NESTIN-expressing neural progenitors, TUJ1-expressing immature neurons, and other cells (S1 Fig). There was no appreciable change in cell density between conditions, consistent with previously published work [29]. Importantly, the timing and duration of modRNA expression matched the known kinetics for other proteins and transcription factors [31]. These data indicate that modRNA transfection enables dose- and time-dependent gene expression in mES-derived cells, including progenitors and neurons.

### Transient Fezf2 expression rescued SCPN fate specification in Fezf2-null mice in vivo

Prior to using *Fezf2* encoded modRNA for transient expression in mES-derived neurons, we first tested the functionality of *Fezf2* modRNA in mice *in vivo*, using an established *Fezf2* null experimental rescue paradigm. In *Fezf2* null mice, SCPN do not develop, and their progenitors are re-specified to CPN [32–34]. However, delivering *Fezf2* by *in utero* plasmid electroporation in *Fezf2*-null mice at E12.5 (when FEZF2 would normally be expressed at a high level) can rescue SCPN specification and their projections to the distal hindbrain [35]. Moreover, at E13.5, E15.5, and later ages, *Fezf2* mis-expression by plasmid electroporation can redirect CPN to acquire most critical features of SCPN identity [32,34,36–38].

We hypothesized that, if transient *Fezf2* expression delivered by *in utero* electroporation of a single dose of *Fezf2* modRNA in Fezf2 null mice can rescue SCPN differentiation, then a similar approach to modRNA delivery might effectively direct differentiation of ES-derived pallial-like progenitors *in vitro*. The extent of *in utero* GFP modRNA electroporation into the pallium (Fig 1C) was largely limited to the mitotic (Ki67-expressing) ventricular zone (Fig 1D). In *Fezf2* null mice at E12.5, we found that *in utero* electroporation of *Fezf2* modRNA (with tdTomato plasmid for long-term visualization of axons; Fig 1E–1G) rescued a small subset of SCPN that project beyond the thalamus to the cerebral peduncle (Fig 1H and 1I). This finding was striking, as multiple control experiments with electroporation of control plasmids (e.g., tdTomato or GFP) did not demonstrate any axonal projections caudal to the striatum, as verified by multiple independent investigators in our laboratory and by other groups [32–34,39,40]. To control for the timing of modRNA expression, *in utero* electroporation of *Fezf2* modRNA to pallial progenitors at a later timepoint, E15.5, did not replicate the *Fezf2* modRNA-mediated SCPN specification or axonal projections (data not shown). These data suggest that a single, transient dose of *Fezf2* can be sufficient to rescue a subset of *Fezf2*-null SCPN, and that this relatively small dose of *Fezf2* modRNA might only be functional within the permissive molecular context of E12.5 neocortical progenitors.

### Transient Fezf2 expression alone did not significantly promote SCPN differentiation by mES-derived neocortical-like neurons

We next asked whether *Fezf2* modRNA alone can induce SCPN-specific differentiation by mES-derived neocortical-like cells. Because the existing protocols of monolayer differentiation [8,22,30] generate limited quantities of mES-derived neocortical neurons, an unbiased approach using randomized, blinded, automated imaging was employed to count sufficient numbers of neocortical-like neurons for these analyses (see Methods and S3 Fig for details). This blinded, automated imaging was performed at 20x magnification, on approximately 20 randomly selected fields per well, sampling approximately 20% of the available surface area, counting > 1,000 cells per well using nuclear staining and *a priori* determined size criteria to assess viability and single cell counts. A high threshold for positive antibody labeling was manually established a priori, blinded, because populations of mES-derived neurons express a continuum of transcription factor labeling intensities, in striking contrast to populations of primary dissociated E12.5 and E15.5 mouse neocortical neurons, which typically display distinct trimodal labeling (negative, low expression, high expression). Using this automated method of cell counting, our baseline protocol of differentiation produced approximately 60% viable nuclei per imaging field based on Hoechst nuclear staining and size criteria, with approximately 10% positive for CTIP2 immunolabeling and approximately 20% positive for SATB2 immunolabeling (see also Sadegh and Macklis, 2014). Normally, induction of CTIP2 expression occurs within 48 hrs of *Fezf2* plasmid expression. However, 48 hrs after *Fezf2* modRNA transfection in mES-derived neocortical cells at *in vitro* day 19, the total numbers of either CTIP2- or SATB2-expressing neocortical neurons were not increased (S2 Fig). This result was not surprising given the heterogeneity and immaturity of neocortical-like neurons in this established protocol of ES cell culture [8].

We hypothesized that, rather than broadly promoting CTIP2 expression in non-neocortical-like neurons, a transient dose of *Fezf2* modRNA might promote SCPN subtype-specific refinement by only a smaller subset of neocortical-like neurons, perhaps those already “poised” to differentiate further into corticofugal neurons. We developed an *a priori* determined metric for delineating SCPN identity refinement in this subset of neocortical-like neurons by quantifying in a blinded manner the ratio of neurons that have matured and only express CTIP2 to neurons that remain immature and have overlapping expression of CTIP2 and SATB2. Even by this more nuanced metric of SCPN identify refinement, we found that *Fezf2* modRNA expression in mES-derived neocortical cells did not, by itself, significantly increase SCPN subtype differentiation (S2 Fig; although there was a trend toward increased SCPN). These data indicate that a single, transient dose of *Fezf2* expression by mES-derived neurons was not sufficient to refine SCPN identity, given the inappropriate molecular context of heterogeneous and maturation-stalled mES-derived neocortical-like neurons (Sadegh and Macklis, 2014), suggesting that additional, potentially complementary manipulations are needed to more optimally direct SCPN differentiation.

### Small molecule screening of mES-derived neocortical-like neurons identified SIRT1 inhibitors

We next asked whether remodeling the chromatin landscape might enable a higher proportion of neocortical-like neurons to respond to *Fezf2*-mediated SCPN subtype refinement. To address this question, we designed an approach combining small molecule screening with transient *Fezf2* induction: 1) directed mES cell differentiation to day 14 neocortical-like progenitors, 2) addition of a small molecule library and incubation for four days to precondition the cells to a more permissive epigenetic landscape, 3) *Fezf2* modRNA transfection and incubation for two days to direct SCPN subtype refinement, and 4) blinded immunocytochemical assessment using multiple exclusionary markers to evaluate the extent of SCPN identity refinement (S3 Fig). We designed a custom library of 80 small molecules modulating known epigenetic enzymes, with targets including histone deacetylases, methyltransferases, and kinases. Using automated, blinded confocal imaging, cell segmentation, and threshold analyses, we quantified the expression of both CTIP2 and SATB2 by individual neurons.

**Fig 2:**
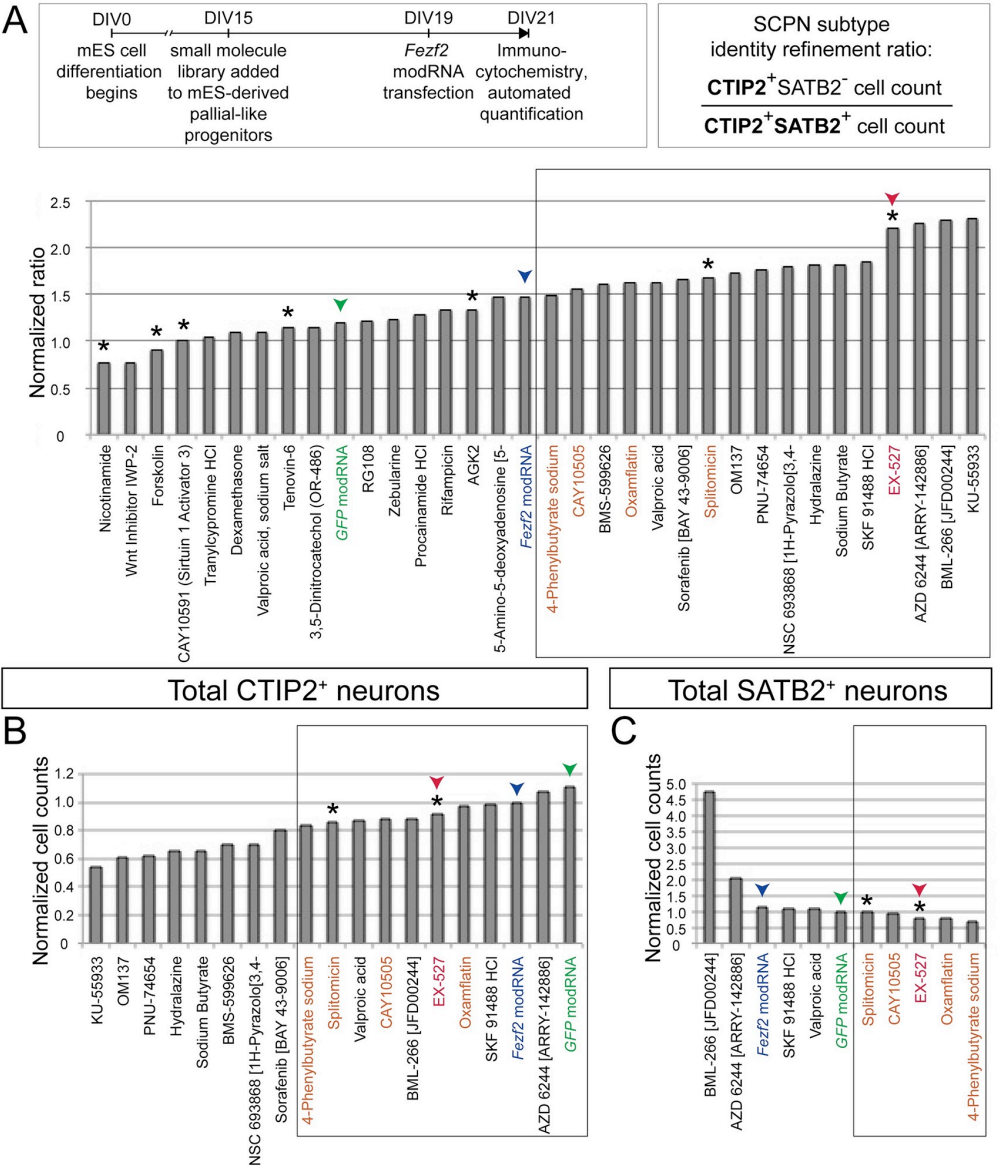
High-content small molecule screen of SCPN/CSN molecular refinement by mES-derived neurons identified candidate small molecule regulators, including the SIRT1 inhibitor EX-527. (A) Each well of a 96-well plate containing ES-derived neurons at 15 days *in vitro* (DIV) was incubated with a distinct small molecule from the library (S3 Fig) at a concentration of 1 micromolar for four days, transfected with *GFP* or *Fezf2* modRNA at 19 DIV with a transfection efficiency of approximately 20%, then incubated for an additional 48 hrs, and analyzed in a blinded manner at 21 DIV. Distinct small molecules enhanced, inhibited, or did not substantially alter *Fezf2*-mediated SCPN subtype refinement, as indicated by the ratio of CTIP2(+)/SATB2(-) neurons to CTIP2(+)/SATB2(+) neurons. The ratios were normalized to the untreated condition. (B) Some candidate small molecules, from the box marked in (A), altered the number of CTIP2-expressing neurons, relative to the *Fezf2* induction condition. A subset of candidates that preserved the highest number of CTIP2+ neurons were chosen for the subsequent analysis. The total CTIP2+ cell counts were normalized to the *Fezf2* modRNA condition. (C) Candidate small molecules, from the box marked in (B), decreased the number of SATB2-expressing neurons relative to *Fezf2* induction alone; these top candidates are enclosed in a new box and highlighted in orange text (in order to identify them in the prior analyses in panels A and B); they include the SIRT1 inhibitor EX-527. The total SATB2+ cell counts were normalized to the *GFP* modRNA condition. Asterisks indicate small molecules that modify HDAC Class III (Sirtuin). Green arrowheads and text color indicate the *GFP* transfection condition and the blue arrowheads and text color indicate the *Fezf2* transfection condition, for comparison. The red arrowheads and text color indicate the EX-527 condition, a relatively specific SIRT1 inhibitor. Data from this first-level, blinded screen experiment (N = 1; the COVID pandemic and shared space use restrictions precluded repetition, though the screen was validated by secondary investigation, thus avoiding false positive results; false negatives might exist) represented approximately 1,000 cells per condition, from 20 randomly sampled fields at 20x magnification.

We used multiple *a priori* selection criteria to identify leading candidates. In the first assay, we found that multiple Sirtuin modulators could either enhance or diminish *Fezf2*-mediated subtype refinement, as indicated by our metric of SCPN subtype identity refinement, hereby defined as the ratio of maturing CTIP2^+^/SATB2^-^ neurons to relatively immature CTIP2^+^/SATB2^+^ double-positive neurons (Fig 2A). Focusing on small molecules that might enhance *Fezf2*-mediated refinement of CTIP2^+^/SATB2^-^ expression, we then asked which small molecules globally increase or maintain the total number of CTIP2 expressing neurons, relative to *Fezf2* modRNA induction alone (Fig 2B). In a third-level assay, we tested a smaller group of leading candidate small molecules for their ability to either maintain or decrease the total number of SATB2 expressing neurons compared with *Fezf2* induction alone (Fig 2C). Optimized by these stringent criteria, and given the prevalence of candidates independently targeting the same Sirtuin pathway, we selected the SIRT1 inhibitor EX-527 as a leading “exemplar” candidate to enhance *Fezf2*-mediated SCPN differentiation. As an internal control, we compared the activity of EX-527 to other Sirtuin inhibitors and activators. Non-specific Sirtuin inhibitors (nicotinamide, forskolin, and tenovin-6) did not increase SCPN refinement. Reinforcing these results, the SIRT1-specific *activator* (CAY10591) displayed antagonism to SCPN refinement.

Since some small molecules were anticipated to produce cellular toxicity, we screened each well in a blinded manner for toxicity prior to performing the above differentiation-focused analyses. Exclusion criteria for further analysis were as follows: evidence for globally reduced cell counts; increased surface area between clusters of cells; excess pyknotic nuclei or cellular debris; or disruption of the normal rosette-like clustering of neocortical-like cells. Of the 80 small molecules tested, only 32 small molecules were included for the analysis shown in Fig 2 and any subsequent experiments. Of the excluded small molecules, 16 produced a moderate degree of toxicity, and 31 produced severe toxicity. Some of these excluded small molecules might warrant future investigation at lower concentrations (replication across dose ranges was precluded by the COVID pandemic and associated shared space restrictions). All data, including cell counts and average intensities of immunostaining for excluded small molecules, are included in the supplementary data (S1 Spreadsheet). While these substantial efforts were made to exclude major sources of cellular toxicity in these analyses, more subtle toxicity limited to a subset of cells that are CTIP2/SATB2 double positive might theoretically contribute to selective refinement and relative increase in CTIP2-only cells. This theoretical possibility is not supported by the literature as a likely one, but warrants future investigation of potential selective toxicity to more mature CTIP2/SATB2 dual-expressing neurons, and/or to other unique neocortical neuron populations.

### SIRT1 inhibition refined primary dissociated E12.5 neuron SCPN subtype identity

**Fig 3:**
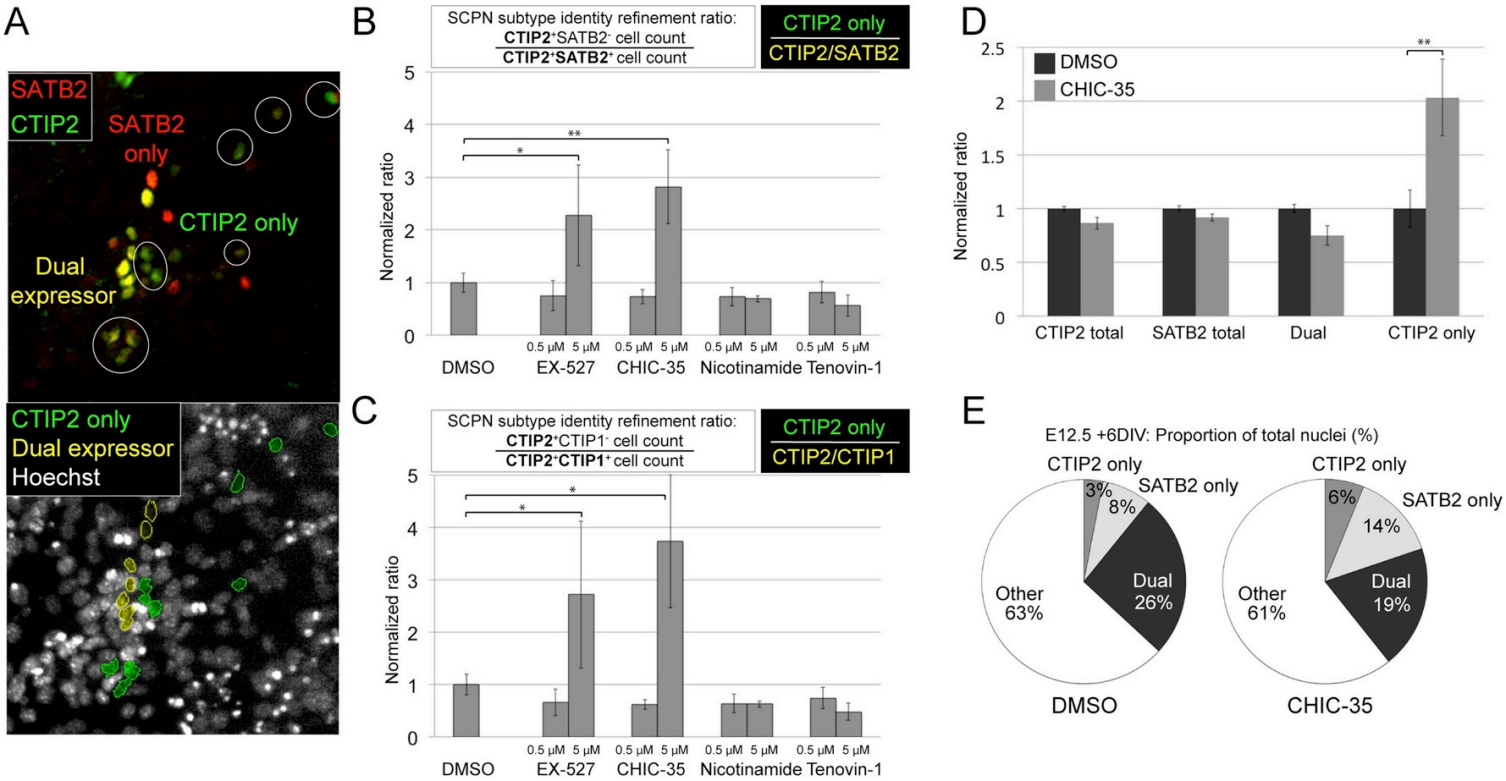
SIRT1 inhibition in dissociated E12.5 neocortical neurons enhanced SCPN/CSN subtype refinement, increasing the number of CTIP2-expressing, SATB2-negative neurons at the expense of CTIP2/SATB2 dual-expressing neurons. (A) Representative image of E12.5 primary dissociated neocortical cells following six days of culture and representative immunostaining with CTIP2 and SATB2. CTIP2+/SATB2- neurons (pseudo-colored green) could be distinguished from CTIP2+/SATB2+ “dual expressor” neurons (pseudo-colored yellow) and counted using our automated imaging and cellular segmentation protocol (S3C–S3E Fig). (B) EX-527 and a more specific SIRT1 inhibitor, CHIC-35, increased CTIP2^+^/SATB2^-^ subtype distinction relative to DMSO-only controls (0.5 micromolar and 5 micromolar). (C) EX-527 and CHIC-35 also trended toward CTIP2^+^/CTIP1^-^ subtype distinction. (D) Importantly, while the proportions of total CTIP2- and total SATB2-expressing neurons did not change relative to DMSO control following CHIC-35 SIRT1 inhibition, the relative proportion of CTIP2/SATB2 dual-expressing neurons decreased. In contrast, the relative proportion of more fully distinguished CTIP2(+)/SATB2(-) neurons increased (reflecting improved SCPN/CSN differentiation). (E) Pie chart schematics show the relative proportions of total nuclei of CTIP2+-only neurons, SATB2+-only neurons, CTIP2+/SATB2+ dual expressing neurons, and unlabeled cells derived from E12.5 neocortical cells in CHIC-35 SIRT1 inhibition treatment conditions versus DMSO control. Data are presented as mean +/- s.e.m. (N = 3; >5,000 nuclei screened per condition from 40 randomly sampled fields at 20x magnification) *P < 0.05; **P < 0.01 (unpaired t-test).

We next asked whether SIRT1 inhibition also promotes SCPN subtype distinction by primary neocortical neurons. In these experiments, *Fezf2* was not induced with modRNA, because *Fezf2* is already highly expressed by primary SCPN progenitors. Dissociated E12.5 neocortical progenitors and neurons were directly treated with small molecule inhibitors of SIRT1 for six days. The total treatment time (six days) matches the total treatment time in the prior experiment using mES-derived culture (four days for small molecule incubation, plus two days for modRNA transfection). At baseline, primary dissociated neocortical neurons demonstrated approximately 20–30% positivity for CTIP2 immunolabeling, and approximately 30–40% positivity for SATB2 immunolabeling; a majority of each immunostained population expressed both CTIP2 and SATB2 (Fig 3A). We again identified EX-527, and an even more specific SIRT1 inhibitor, CHIC-35, as potent enhancers of CTIP2^+^/SATB2^-^ subtype identity refinement (Fig 3B). CHIC-35 is highly SIRT1-specific, with a binding site within the SIRT1 catalytic cleft that blocks substrate binding [41,42]. Compared to non-specific inhibitors, both EX-527 and CHIC-35 show selective enhancement of SCPN molecular refinement (Fig 3B). Notably, there was no observable change in the density of cultured cells following small molecule application, providing evidence for absence of substantial toxicity, which was confirmed by automated, blinded cell segmentation analysis demonstrating stable cell counts, sizes, and fluorescence in the imaging wells across conditions.

To further investigate whether SIRT1 inhibition broadly regulates SCPN subtype identity, rather than potentially only downregulating SATB2 expression, we asked whether other subtype-specific refinements occur. CTIP1 is a transcription factor that regulates both subtype- and area-specific identity [10,11]. Despite its close homology to CTIP2, CTIP1 is initially co-expressed with CTIP2, but its expression later becomes excluded from SCPN, restricted to CPN and corticothalamic projection neurons, and is largely limited to expression in primary sensory areas. We found that the strategy of small molecule SIRT1 inhibition additionally promoted CTIP2^+^/CTIP1^-^ subtype distinction (Fig 3C). Based on both CTIP2^+^/SATB2^-^ and CTIP2^+^/CTIP1^-^ subtype distinction in the context of *Fezf2* expression, these data indicate that SIRT1 inhibition enhances and enables *Fezf2* refinement of neocortical subtype identity toward SCPN.

Given the known roles of SIRT1 in cortical neural progenitor differentiation and neuronal survival [19,43–48] and post-mitotic cortical neuron genomic stability [49], we next tested an alternative theoretical hypothesis that SIRT1 inhibition might potentially alter the proportions of progenitors and neurons, giving an impression of post-mitotic subtype distinction, while instead acting at the progenitor level. To the contrary, we found that the increase in proportion of CTIP2-expressing neurons was nearly evenly compensated by the reduction in number of CTIP2^+^/SATB2^+^ dual-expressing neurons (Fig 3D and 3E). Because the combined fraction of CTIP2- and SATB2-expressing neurons remained constant between samples, these experiments indicated that the subtype refinement phenotype was not due to changes in the proliferation of neocortical progenitors.

Although EX-527 and CHIC-35 are highly specific small molecule inhibitors of SIRT1 [42], we pursued *Sirt1*-specific molecular knockdown with siRNA to further investigate and test whether SIRT1 is the main target of repression in primary dissociated neocortical neurons. We found that *Sirt1* knockdown in primary dissociated E12.5 neocortical neurons recapitulated the effect of small molecule inhibition of SIRT1, increasing both CTIP2^+^/SATB2^-^ and CTIP2^+^/CTIP1^-^ subtype-specific SCPN identity refinements (S4 Fig).

### Subtype-specific SIRT1 expression during embryonic and postnatal development

**Fig 4:**
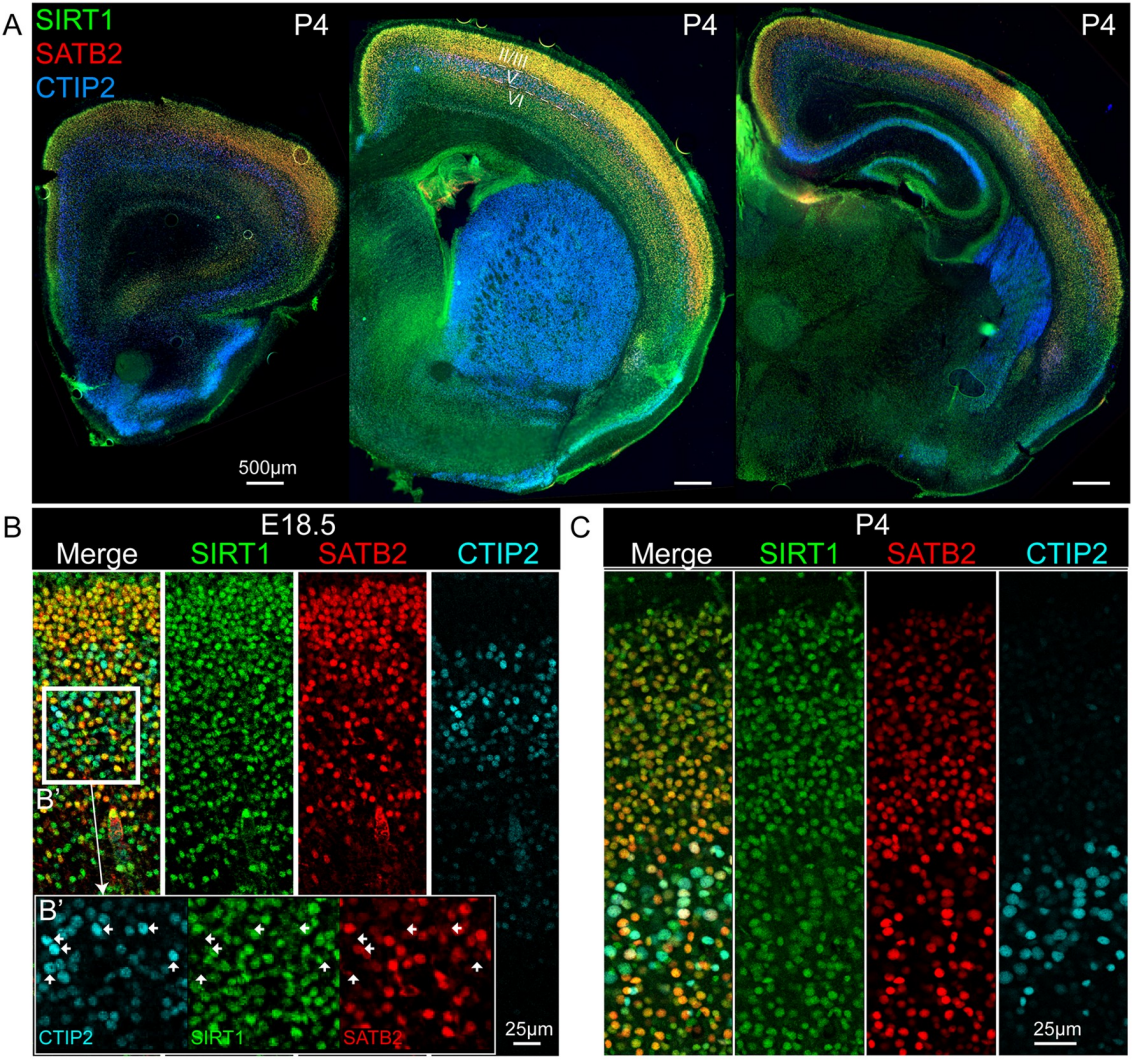
SIRT1 was differentially expressed by CPN and SCPN/CSN *in vivo*. (A) At P4, immunocytochemical labeling showed that SIRT1 was expressed along the entire rostral-caudal extent of the neocortex, in layers II/III, VI, and subplate (50 micrometer coronal section, wide-field fluorescence imaging). (B) At E18.5, SIRT1 expression in deep layers of motor cortex was predominantly restricted to SATB2-expressing neurons, and was absent or expressed at quite low levels by CTIP2-expressing neurons (50 micrometer coronal section, confocal fluorescence imaging). (C) At P4, SIRT1 expression in deep layers of motor cortex was almost completely restricted to SATB2-expressing neurons (50 micrometer coronal section, confocal fluorescence imaging).

We next investigated whether *in vivo* SIRT1 expression is consistent with the results of the screening approach in mES-derived neurons. We used immunocytochemistry to investigate SIRT1 protein localization in the developing mouse neocortex. At P4, we found that SIRT1 is expressed throughout the rostro-caudal extent of the neocortex, in layers II/III, V (at a relatively lower level), VI, and subplate (Fig 4A). While SIRT1 expression is broadly distributed, as previously reported [50–52], we hypothesized that its level of expression varies in distinct neocortical subtypes. Strikingly, we found that SIRT1 expression was subtype-specific by E18.5, with near complete co-localization with SATB2-expressing CPN in layers II/III, V, and VI, and exclusion by CTIP2-expressing SCPN/CSN in layer V (Fig 4B). Similarly, at P4, SIRT1 expression was excluded from increasingly mature SCPN/CSN (Fig 4C).

We next asked whether SIRT1 is differentially transcribed in pure populations of retrogradely-labeled CPN versus CSN, the important subtype of SCPN in layer V that project axons to the spinal cord. Using an existing microarray-based comparative gene expression analysis of retrogradely-labeled CPN and CSN [39,53], we found that SIRT1 is the only differentially expressed histone deacetylase (HDAC) throughout post-mitotic neocortical differentiation at E18.5, P3, P6, and P14, with highest expression by CPN at all ages (S5 Fig). Combined with the protein expression data in Fig 4, these results demonstrated that *Sirt1* mRNA and protein are specifically and highly expressed by SATB2-expressing CPN subtypes during corticogenesis, and are expressed at significantly lower levels by CTIP2-expressing SCPN/CSN during early, middle, and late stages of subtype identity refinement. These findings support the CPN-specific expression of SIRT1 during development, and its relative exclusion from SCPN/CSN and other neocortical neurons.

Together, these data identify a context-specific role for SIRT1 in partially refining SCPN/CSN post-mitotic identity refinement during late embryonic neocortical development. Identified by high content, small molecule screening of epigenetic factors, SIRT1 inhibition enhanced SCPN/CSN molecular refinement among primary dissociated E12.5 neocortical neurons, and complemented the approach of using transient *Fezf2* modRNA expression to promote SCPN/CSN identity refinement among heterogeneous mES-derived neocortical-like neurons.

## Discussion

Our data indicate that *Sirt1* inhibition or knockdown approaches are effective for the refinement of ES-derived SCPN/CSN *in vitro*, and suggest that SIRT1 is functionally important for the refinement of SCPN/CSN identity *in vivo* during mouse neocortical development. This is likely also relevant for human iPSC directed differentiation into SCPN/CSN. From the initial screening experiment (Fig 2), we found that inhibition of SIRT1 preceding *Fezf2* induction enhanced SCPN/CSN identity refinement in mES-derived neocortical neurons. Within primary mouse neocortical neurons, we identified that *Sirt1* inhibition, by either small molecule or knockdown approaches, promoted mature molecular refinement of *Fezf2*-mediated SCPN/CSN identity (Figs 3 and S4 Fig). Although *Sirt1*-null mice have not yet been assessed for subtype-specific deficits in the neocortex, their gross neocortical anatomy (*e*.*g*. intact corpus callosum, absence of Probst bundles) appears intact [54–56], suggesting that *Sirt1* is not required for CPN specification.

SIRT1 is a ubiquitously expressed NAD-dependent histone deacetylase (HDAC) with context-dependent roles in neocortical differentiation [19,45]. At early developmental stages, SIRT1 regulates neurogenesis within neocortical progenitors by repressing the Notch-Hes pathway [19]. Later in development, SIRT1 is ubiquitously expressed, with minimal enrichment in the upper layers of mouse neocortex at E14.5 and at 10 months of age, although the level of SIRT1 expression by specific neocortical subtypes had not been previously assessed [50–52]. We find that neocortical SCPN/CSN have markedly reduced SIRT1 expression in mid- to late-corticogenesis, in contrast to deep and superficial layer CPN that are relatively enriched for SIRT1 expression (Figs 4 and S5 Fig). Based on these results, SIRT1 should be considered to have neuronal subtype specificity as a chromatin modifier in the neocortex [14,32,53].

Multiple lines of evidence indicate that SCPN/CSN identity refinement (*in vivo* and in mES-derived neurons) requires both *Fezf2* and a permissive molecular context during differentiation. First, transient *Fezf2* expression is sufficient to generate SCPN/CSN in *Fezf2*-null mice at E12.5, but not at E15.5 (Fig 1). At later ages (E13.5 through P7), a higher dose or duration of *Fezf2* expression (e.g., by plasmid vector) can re-specify alternate neocortical subtypes toward most aspects of SCPN/CSN identity [32,34,37,38]. However, after E15.5, mis-expression of *Fezf2* does not induce Ctip2 expression in most neurons, highlighting context specificity [34,37,38]. Together, these prior *in vivo* findings indicate that E12.5 is the optimal molecular context to most completely enable *Fezf2*-mediated specification of SCPN, induction of Ctip2 expression, and stable epigenetic silencing of Satb2. Consistently, *in vitro*, high dose *Fezf2* induction (plasmid or viral mediated) by neocortical-like neurons does not alone induce SCPN/CSN identity [27]. Moreover, when *Fezf2* modRNA is induced within mES-derived neocortical cells at a time approximating E12.5 neocortical differentiation, it alone does not significantly increase SCPN/CSN subtype-specific transcription factor expression (S2 Fig). These *in vitro* data indicate that, although the timing of *Fezf2* expression is important, SCPN/CSN-directed differentiation cannot be accomplished by targeting cells with a suboptimal chromatin landscape, as would be expected in maturation-stalled ES-derived neocortical neurons [8]. We reasoned that *Fezf2* requires a favorable epigenetic context in order to optimally transcribe its target genes, so we designed all experiments such that SIRT1 would be delivered earlier or at the same time as *Fezf2*. Because SIRT1 itself is unlikely to provide SCPN-specificity in the absence of Fezf2, we did not pursue experiments with the reverse order of reagent addition (i.e. *Fezf2* before SIRT1) in mES-derived cell culture.

CPN are an evolutionarily more recent and diversified subtype of neocortical neurons, and likely employ multiple sequential epigenetic mechanisms in their specification, molecular refinement, and maturation [13–17,53,57–60]. At late stages of maturation of layer 2/3 CPN (*e*.*g*. eight postnatal weeks in mice), the widely expressed methyl binding protein MeCP2 is required for proper development and/or maintenance of dendritic complexity and soma size [13,57,61]. At earlier stages of CPN development, SATB2 is required for proper differentiation, indirectly guiding chromatin remodeling by binding to matrix attachment regions (MAR) to mediate long-range interactions of enhancer sites with promoters [62–64], including the recruitment of HDAC enzymes through a binding partner, SKI [5,6,65–67]. Together–with varying extents of CPN-specificity–SIRT1, SATB2/SKI, MeCP2, and likely others [68] might coordinate chromatin remodeling in CPN at distinct stages of development.

Moreover, the transcription factors CTIP2 and CTIP1 (BCL11B, BCL11A; [69]), which are differentially expressed with subtype-specificity in the cortex, and which regulate the precision of SCPN and CPN differentiation [10,11,39,70,71] have also been demonstrated to interact with both the NuRD complex [72,73] and SIRT1 [74,75] to mediate chromatin remodeling in cells outside of the brain. Together, these reports are consistent with our data supporting SIRT1-mediated contributions to post-mitotic refinement of CTIP2-expressing SCPN from SATB2-/CTIP1-expressing CPN.

The postmitotic subtype-specificity of SIRT1 expression in the neocortex is also remarkable because SIRT1 is implicated in the oxidative stress response and survival of neurons [43,44]. Because SIRT1-expressing CPN might be resistant to metabolic insults, it raises the possibility that SCPN/CSN, by virtue of having reduced SIRT1 expression, might be more sensitive to metabolic stress. SCPN and especially the subpopulation of corticospinal neurons (CSN) are the brain neurons that selectively degenerate in amyotrophic lateral sclerosis (ALS; [76,77]). Intriguingly, *Sirt1* over-expression has been shown to promote short-term survival of dissociated neocortical neurons mis-expressing ALS associated mutant SOD1 [78]. More broadly, non-specific HDAC inhibitors show neuroprotective properties in mouse models of ALS [79,80].

Overall, our findings indicate subtype-specific functions for SIRT1 in the molecular refinement of neocortical SCPN/CSN versus CPN identity. Importantly, these results demonstrate the utility of combining epigenetic priming with subtype-specific transcription factor induction in ES (and not unlikely, human iPSC) directed differentiation. These results provide a proof-of-concept strategy for specific and progressive enhancement of directed CSN/SCPN or other subtype differentiation from pluripotent cells. These results further suggest that subtype-specific epigenetic modulation might enhance optimal *in vitro* generation of other diverse neocortical neuron subtypes.

## Materials and methods

Animal Research approved by the Harvard Institutional Animal Care and Use Committee. Euthanasia performed as per institutional guidelines.

### Mice

*Fezf2*-null mice were the generous gift of S. McConnell [33]. Wild-type CD1 mice were used in all other experiments (Charles River Laboratories). The date of vaginal plug detection was designated E0.5, and the day of birth as P0. Sex was not used as a selection criterion for any experiment. Mice were euthanized by deep anesthesia with Avertin followed by transcardial perfusion with 0.1 M phosphate-buffered saline (PBS), then with 4% paraformaldehyde (PFA) in PBS buffer. Brains were postfixed in 4% PFA overnight and sectioned with a vibrating microtome (Leica). Mice used in these experiments were handled according to guidelines of the National Institutes of Health (NIH), and all procedures were conducted in accordance with Harvard University’s institutional guidelines.

### Immunocytochemistry

Primary antibodies and dilutions used were: rat antibody to CTIP2 (1:500, Abcam); mouse antibody to SATB2 (1:200, Abcam); rabbit antibody to SIRT1 (1:250, Millipore); rabbit antibody to CTIP1 (1:500, Abcam); rabbit antibody to GFP (1:500, Invitrogen); rabbit antibody to Ki67 (1:500, Abcam); chicken antibody to NESTIN (1:500, Novus Biologicals); mouse antibody to TuJ1 (1:500, Covance); and mouse antibody to MAP2 (1:500, Sigma). Alexa fluorophore conjugated secondary antibodies from Invitrogen were used at a dilution of 1:1000. Hoechst 33342 counterstain was used to visualize nuclei (1:3,000, Invitrogen).

### RNA synthesis and transfection

Synthetic modified RNA (modRNA) was generated as described [29]. Briefly, RNA was synthesized with the MEGAscript T7 kit (Ambion, Austin, TX). A custom ribonucleoside blend was used, comprising 6 mM 5’ cap analog (New England Biolabs), 7.5 mM adenosine triphosphate and 1.5 mM guanosine triphosphate (USB, Cleveland, OH), 7.5 mM 5-methylcytidine triphosphate and 7.5 mM pseudo-uridine triphosphate (TriLink Biotechnologies, San Diego, CA). Transfections of modRNA and multiple siRNA targeted against *Sirt1* and *Satb2* (both from Santa Cruz) were carried out with RNAiMAX (Invitrogen), as per the manufacturer’s instructions.

### Cell culture and differentiation

Feeder-free E14Tg2a (Baygenomics) mouse embryonic stem cells (mES) were passaged on gelatin-coated (0.1% gelatin, StemCell Technologies) cell culture treated plastic dishes using established media and cell culture techniques [8]. For differentiation, mES were plated at low density (5,000 cells / cm^2^) on gelatin-coated plastic dishes in ESC medium, and cultured as described [30]. Cyclopamine (Calbiochem) was added from day 2 to day 10 in the differentiation medium at a final concentration of 1 micromolar. After 10 to 14 days of differentiation, cells were trypsinized, dissociated, and plated on poly-lysine/laminin (Becton-Dickinson) coated glass coverslips, and allowed to grow for 4–14 days in N2B27 medium [30]. Primary culture of E12.5 neocortical neurons was performed as previously described [8] and subsequently cultured in N2B27 medium [30].

### High-content small molecule screening

A high content screening protocol was adapted from Makhortova *et al*. [81]. Briefly, mES were seeded at 5,000 cells per well in 96-well plates, and treated in duplicate at 10 micromolar, 1 micromolar, and 0.1 micromolar with individual compounds from the screening library, a custom set of 80 chemicals affecting histone deacetylases, methyltransferases, and kinases. Most small molecules, including EX-527 (Sigma), CHIC-35 (Sigma), and nicotinamide (Sigma), were re-suspended in DMSO, according to the manufacturer’s instructions. The final dilution of DMSO was 1: 1,000 in ES cell and primary neuron cultures and included in all control conditions with no notable differences in ES cell differentiation at that concentration. Treatments were performed systematically, and analyses were performed in a manner blind to experimental conditions.

96-well plates were scanned by an automated confocal microscope (PerkinElmer Opera) at 20X magnification with separate fluorescence exposures from a UV light source and 488, 546, and 647nm lasers. Image analysis was performed in a blinded manner using Columbus software (version 2.3.0; PerkinElmer; see also S3 Fig, panel C-E), which automatically set the boundaries of cell nuclei based on Hoechst staining. These boundaries were optimized by manual inspection to exclude nuclear fragments or adjacent double nuclei based on the total area and staining intensity of Hoechst-positive nuclei. Next, the intensity of antibody labeling for each distinct transcription factor in each nucleus was quantified. The threshold for positive antibody labeling was manually established *a priori*, individually for CTIP2, SATB2, and CTIP1, compared to baseline labeling without primary antibody (omission of primary controls). This threshold for labeling a nucleus positive for individual transcription factor expression was calibrated to approximately 20% of the maximum average pixel intensity observed in other nuclei for that transcription factor. Setting this threshold for positive labeling was necessary because populations of mES-derived neurons express a near-continuum of transcription factor labeling intensities, creating a large population of cells with simultaneous faint nuclear expression of multiple transcription factors, which could be interpreted as indicative of atypical transcriptional regulation (“confused” neurons). This is in striking contrast with the expression levels exhibited by *bona fide* populations of primary dissociated E15.5 mouse neocortical neurons, using the same labeling and analysis methods. These primary neurons typically display much more distinctly grouped average pixel intensities for each nucleus, with largely trimodal labeling that can be quantitatively delineated (negative, low expression, high expression).

## Supporting information

S1 Fig modRNA transfection was not biased to a specific neural population. (A) At 24 hours, *GFP* modRNA transfected mES-derived neocortical-like cells at 19 DIV expressed GFP within progenitors (NESTIN-expressing, empty arrows), immature neurons (TuJ1-expressing, filled arrows), and other cells. Approximately 20% of cells are transfected with GFP. (B) GFP was expressed by NESTIN-positive mES-derived cells as early as three hours following transfection with *GFP* modRNA. (C) *mCherry* and *GFP* modRNA were co-expressed by the same mES-derived cells 24 hours following transfection (native fluorescence).
(TIF)

S2 Fig *Fezf2* induction in ES-derived neurons did not on its own significantly increase subtype distinction. (A) At 21 days *in vitro* (DIV), the ratio of CTIP2+/SATB2- neurons to CTIP2+/SATB2+ dual expressing mES-derived neurons was not statistically significantly increased 48hrs after *Fezf2* and *GFP* modRNA co-transfection (dark grey) relative to *GFP* modRNA transfection alone (light gray) (though a trend suggests potentially modest increase of approximately 20%). The total number of CTIP2-expressing neurons was largely unaffected, as was the number of total SATB2-expressing and CTIP2+/SATB2+ dual expressing neurons (though the latter two displayed non-statistically significant trends toward decrease in number). (B) The intensity of CTIP2 expression within *Fezf2*, *GFP* modRNA co-transfected neurons increased relative to *GFP* modRNA controls. Data are presented as mean +/- s.e.m. (N = 2; approximately 1,000 cells per condition, from 20 randomly sampled fields at 20x magnification).
(TIF)

S3 Fig Design of high throughput small molecule screening protocol to identify potential regulators of subtype refinement within cortical-like neurons. (A) Schematic of screening strategy in 96-well plates. Monolayer mES differentiation to telencephalic progenitors was followed by the addition of a custom small molecule library; the composition of this library is described in (B). After small molecule incubation for four days, each well was transfected with *Fezf2* modRNA. Two days after transfection, cells were fixed and immunolabeled for CTIP2, SATB2, and CTIP1. Automated imaging and fluorescence intensity thresholding algorithms distinguished and counted neurons; an example of this is shown in (C). (B) The composition of a custom set of 80 chemicals regulating histone deacetylases, methyltransferases, and kinases is depicted in this pie chart and found in the supplementary spreadsheet. (C) Columbus software parameters to find nuclei: Common Threshold = 0.1; Area > 10 micrometer^2^; Split Factor = 5.0; Individual Threshold = 0.5; Contrast = 0.05. Parameters to select for viable nuclei: Object Area > 33 micrometer^2^; < 100 micrometer^2^; Object Width > 3.7 micrometer; Mean Hoechst nuclear intensity < 900. With these criteria, 182 viable nuclei were selected out of 317 candidate nuclei in this representative 20X field. (D) Subsets of immunolabeled nuclei were identified by mean pixel intensity. CTIP2-low expressors were selected with mean intensity values between 50 and 150; CTIP2-high expressors had mean intensity values between 150 and 700. SATB2-low expressors were selected with mean intensity values between 150 and 500; SATB2-high expressors had mean intensity values between 500 and 1500. CTIP1-low expressors were selected with mean intensity values between 50 and 90; CTIP1-high expressors had mean intensity values between 90 and 400. (E) Representative CTIP2 immunolabeling is shown, with subsets of positive cells exhibiting high and low mean intensities outlined. Using these parameters, we performed automated and unbiased counting of CTIP2 and SATB2 positive nuclei and also distinguished subsets of cells with multiple markers.
(TIF)

S4 Fig siRNA knockdown of *Sirt1* recapitulates the effect of small molecule inhibition of SIRT1. Left panel: CTIP2/SATB2 refinement increased with *Sirt1* knockdown (KD) in primary dissociated E12.5 neurons co-transfected with *GFP* modRNA, as compared to alternate conditions (co-transfection of *Ngn2* and *GFP* modRNA, or co-transfection of *Satb2* siRNA with *GFP* modRNA) following six days of culture. Right panel: Consistent with multiple molecular refinements during neocortical projection neuron subtype distinction, CTIP2/CTIP1 refinement also increased with *Sirt1* knockdown. Data are presented as mean +/- s.e.m. (N = 3; approximately 5,000 nuclei screened per condition, from 40 randomly sampled fields at 20x magnification). *P < 0.05 (unpaired t-test).
(TIF)

S5 Fig Sirt1 mRNA expression is CPN-specific in the neocortex at late embryonic and postnatal ages. Left panel: *Sirt1* mRNA expression was higher by CPN (red lines) than by CSN (blue lines) at E18.5, P3, P6, and P14; these populations were retrogradely labeled and purified by fluorescence-activated cell sorting (FACS) for comparative gene expression analysis at each time-point (data from [39]). Middle and right panels: Other HDACs (*e*.*g*., *HDAC1* and *HDAC2*) are not differentially expressed at all ages, using the same microarray data.
(TIF)

S1 Spreadsheet Data tables for small molecule library screened in Fig 2, including identity of small molecules, cell counts per imaging field, and average intensities of immunostaining all small molecules. (XLSX)
