# Supplementary figures and images for: Synthetic modified Fezf2 mRNA (modRNA) with concurrent small molecule SIRT1 inhibition enhances refinement of cortical subcerebral/corticospinal neuron identity from mouse embryonic stem cells
Source: PLoS One. 2021 Sep 2;16(9):e0254113. doi: 10.1371/journal.pone.0254113 (PMC8412356; doi:10.1371/journal.pone.0254113)

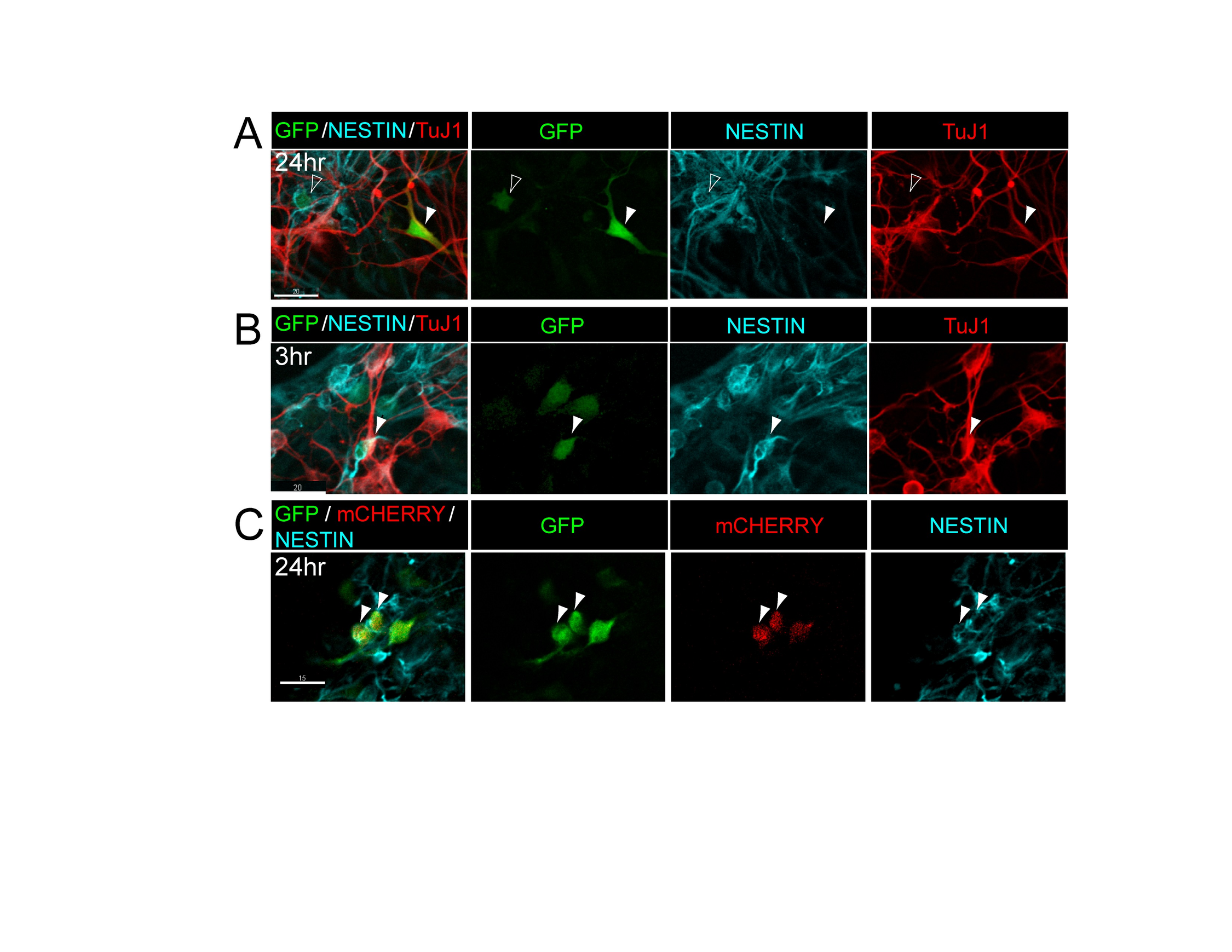

Supplement: S1 Fig — (A) At 24 hours, GFP modRNA transfected mES-derived neocortical-like cells at 19 DIV expressed GFP within progenitors (NESTIN-expressing, empty arrows), immature neurons (TuJ1-expressing, filled arrows), and other cells. Approximately 20% of cells are transfected with GFP. (B) GFP was expressed by NESTIN-positive mES-derived cells as early as three hours following transfection with GFP modRNA. (C) mCherry and GFP modRNA were co-expressed by the same mES-derived cells 24 hours following transfection (native fluorescence). (TIF) [file pone.0254113.s001.tif]

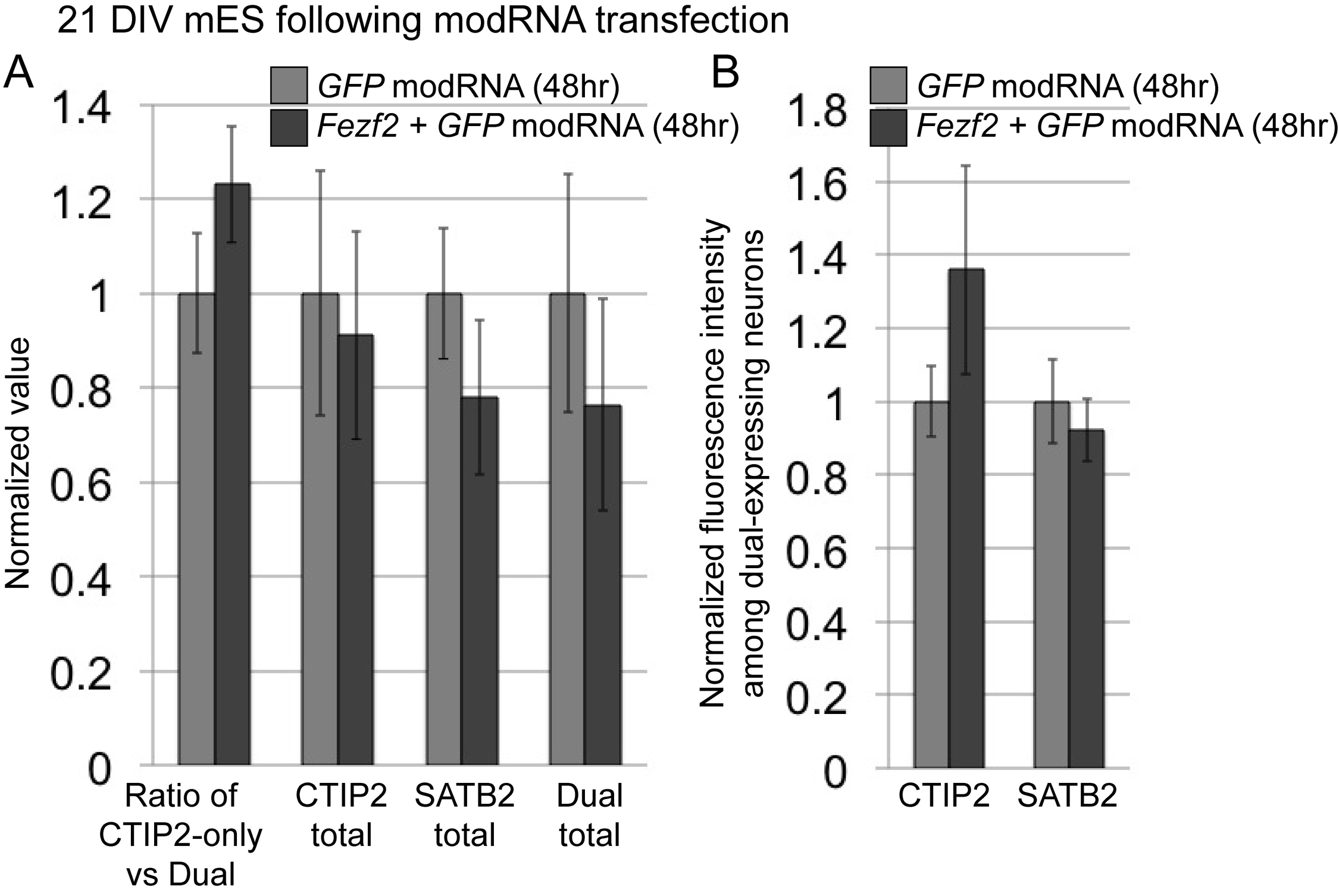

Supplement: S2 Fig — (A) At 21 days in vitro (DIV), the ratio of CTIP2+/SATB2- neurons to CTIP2+/SATB2+ dual expressing mES-derived neurons was not statistically significantly increased 48hrs after Fezf2 and GFP modRNA co-transfection (dark grey) relative to GFP modRNA transfection alone (light gray) (though a trend suggests potentially modest increase of approximately 20%). The total number of CTIP2-expressing neurons was largely unaffected, as was the number of total SATB2-expressing and CTIP2+/SATB2+ dual expressing neurons (though the latter two displayed non-statistically significant trends toward decrease in number). (B) The intensity of CTIP2 expression within Fezf2, GFP modRNA co-transfected neurons increased relative to GFP modRNA controls. Data are presented as mean +/- s.e.m. (N = 2; approximately 1,000 cells per condition, from 20 randomly sampled fields at 20x magnification). (TIF) [file pone.0254113.s002.tif]

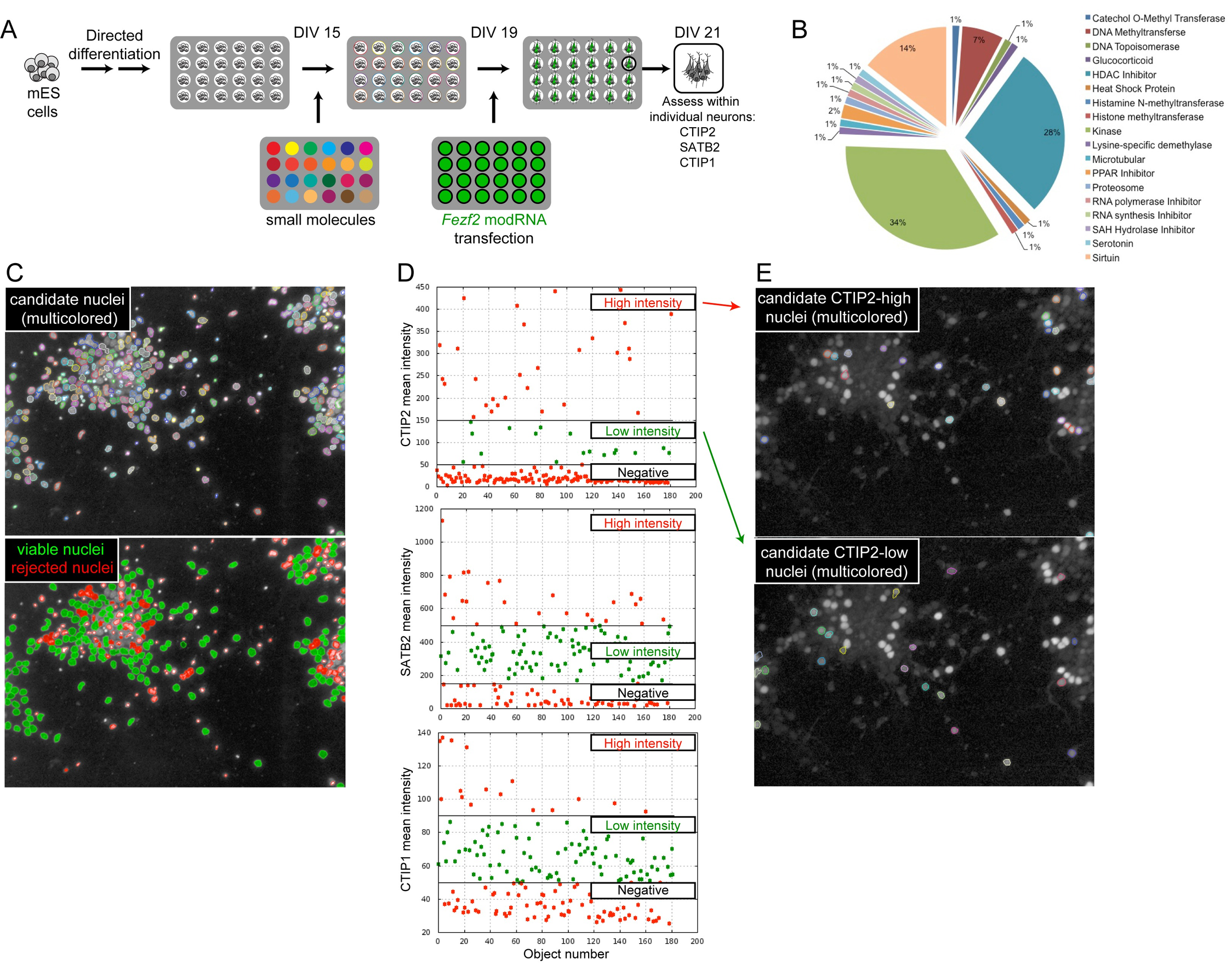

Supplement: S3 Fig — (A) Schematic of screening strategy in 96-well plates. Monolayer mES differentiation to telencephalic progenitors was followed by the addition of a custom small molecule library; the composition of this library is described in (B). After small molecule incubation for four days, each well was transfected with Fezf2 modRNA. Two days after transfection, cells were fixed and immunolabeled for CTIP2, SATB2, and CTIP1. Automated imaging and fluorescence intensity thresholding algorithms distinguished and counted neurons; an example of this is shown in (C). (B) The composition of a custom set of 80 chemicals regulating histone deacetylases, methyltransferases, and kinases is depicted in this pie chart and found in the supplementary spreadsheet. (C) Columbus software parameters to find nuclei: Common Threshold = 0.1; Area > 10 micrometer2; Split Factor = 5.0; Individual Threshold = 0.5; Contrast = 0.05. Parameters to select for viable nuclei: Object Area > 33 micrometer2; < 100 micrometer2; Object Width > 3.7 micrometer; Mean Hoechst nuclear intensity < 900. With these criteria, 182 viable nuclei were selected out of 317 candidate nuclei in this representative 20X field. (D) Subsets of immunolabeled nuclei were identified by mean pixel intensity. CTIP2-low expressors were selected with mean intensity values between 50 and 150; CTIP2-high expressors had mean intensity values between 150 and 700. SATB2-low expressors were selected with mean intensity values between 150 and 500; SATB2-high expressors had mean intensity values between 500 and 1500. CTIP1-low expressors were selected with mean intensity values between 50 and 90; CTIP1-high expressors had mean intensity values between 90 and 400. (E) Representative CTIP2 immunolabeling is shown, with subsets of positive cells exhibiting high and low mean intensities outlined. Using these parameters, we performed automated and unbiased counting of CTIP2 and SATB2 positive nuclei and also distinguished subsets of cells with multipl [file pone.0254113.s003.tif]

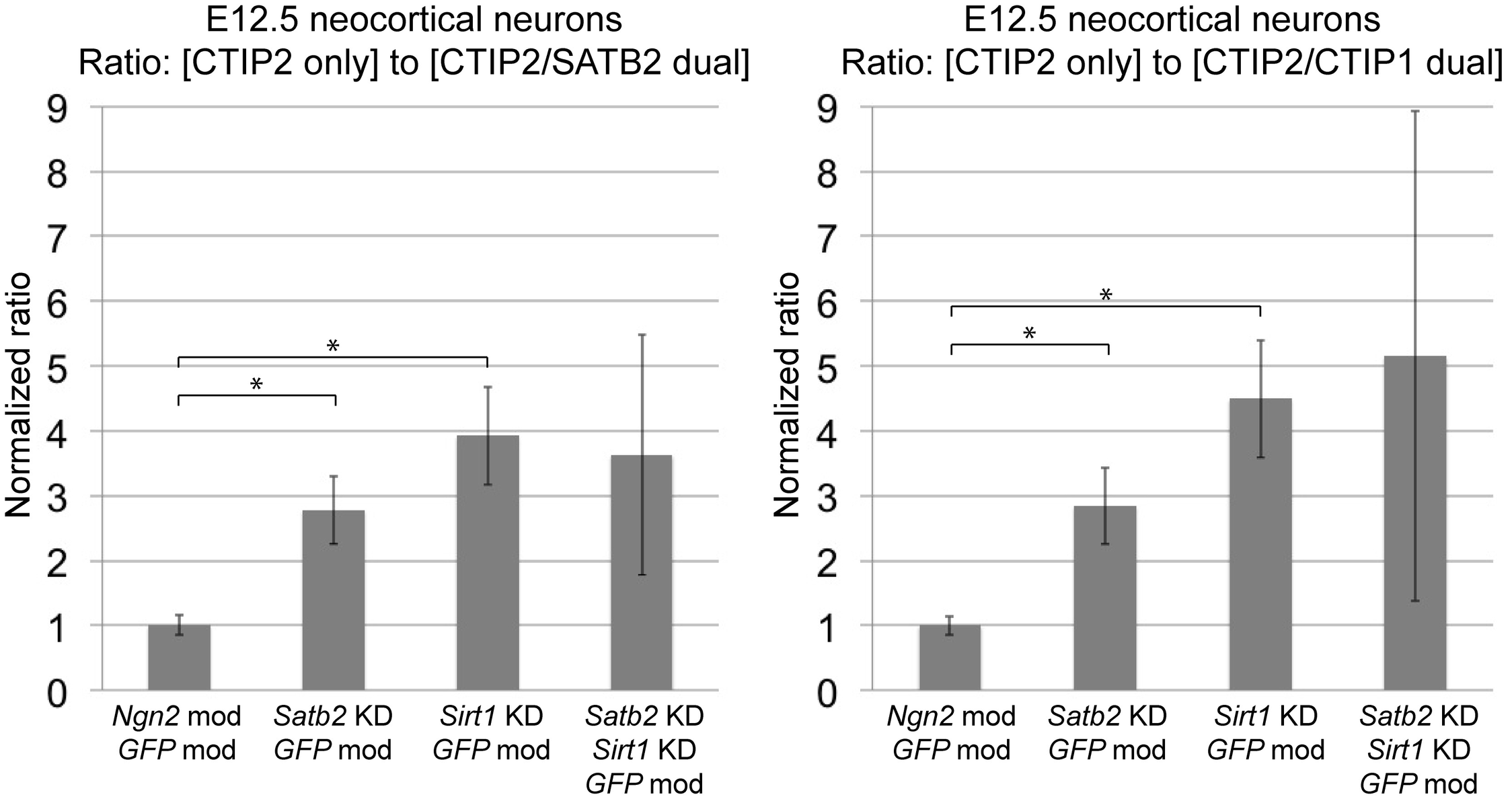

Supplement: S4 Fig — Left panel: CTIP2/SATB2 refinement increased with Sirt1 knockdown (KD) in primary dissociated E12.5 neurons co-transfected with GFP modRNA, as compared to alternate conditions (co-transfection of Ngn2 and GFP modRNA, or co-transfection of Satb2 siRNA with GFP modRNA) following six days of culture. Right panel: Consistent with multiple molecular refinements during neocortical projection neuron subtype distinction, CTIP2/CTIP1 refinement also increased with Sirt1 knockdown. Data are presented as mean +/- s.e.m. (N = 3; approximately 5,000 nuclei screened per condition, from 40 randomly sampled fields at 20x magnification). *P < 0.05 (unpaired t-test). (TIF) [file pone.0254113.s004.tif]

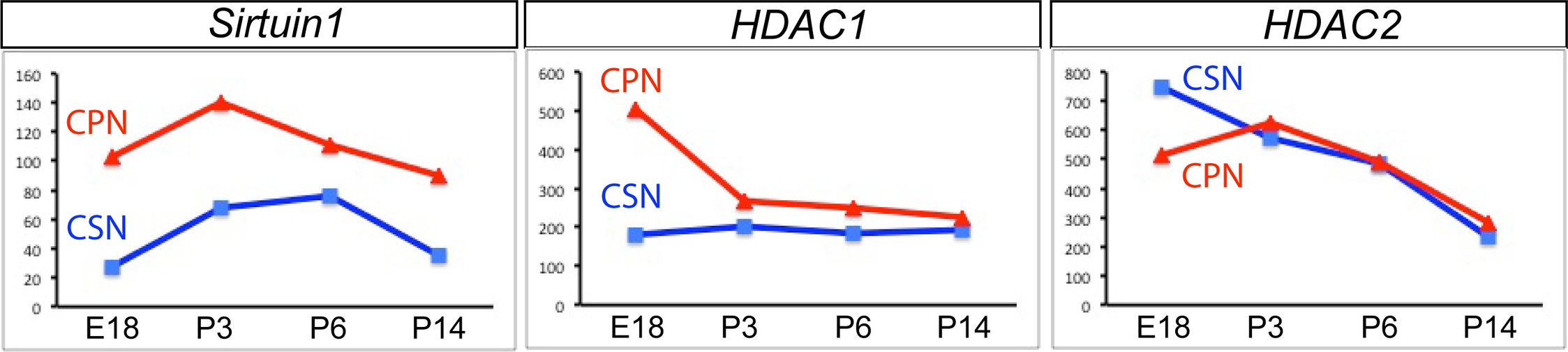

Supplement: S5 Fig — Left panel: Sirt1 mRNA expression was higher by CPN (red lines) than by CSN (blue lines) at E18.5, P3, P6, and P14; these populations were retrogradely labeled and purified by fluorescence-activated cell sorting (FACS) for comparative gene expression analysis at each time-point (data from [39]). Middle and right panels: Other HDACs (e.g., HDAC1 and HDAC2) are not differentially expressed at all ages, using the same microarray data. (TIF) [file pone.0254113.s005.tif]
